# Educational Interventions for Teaching Evidence-Based Practice to Undergraduate Nursing Students: A Scoping Review

**DOI:** 10.3390/ijerph17176351

**Published:** 2020-08-31

**Authors:** Athina E. Patelarou, Enkeleint A. Mechili, María Ruzafa-Martinez, Jakub Dolezel, Joanna Gotlib, Brigita Skela-Savič, Antonio Jesús Ramos-Morcillo, Stefano Finotto, Darja Jarosova, Marta Smodiš, Daniela Mecugni, Mariusz Panczyk, Evridiki Patelarou

**Affiliations:** 1 Department of Nursing, Faculty of Health Sciences, Hellenic Mediterranean University, 71414 Crete, Greece; athina.patelarou@gmail.com (A.E.P.); epatelarou@hmu.gr (E.P.); 2 Clinic of Social and Family Medicine, School of Medicine, University of Crete, 70013 Crete, Greece; 3 Department of Health Care, Faculty of Public Health, University of Vlora, 9401 Vlora, Albania; 4 Faculty of Nursing, University of Murcia, Campus de Espinardo, 30100 Murcia, Spain; maruzafa@um.es (M.R.-M.); ajramos@um.es (A.J.R.-M.); 5 Department of Nursing and Midwifery, Faculty of Medicine, University of Ostrava, 703 00 Ostrava, Czech Republic; jakub.dolezel@osu.cz (J.D.); darja.jarosova@osu.cz (D.J.); 6 Department of Education and Research in Health Sciences, Faculty of Health Sciences, Medical University of Warsaw, 02-091 Warsaw, Poland; joanna.gotlib@wum.edu.pl (J.G.); mariusz.panczyk@wum.edu.pl (M.P.); 7 Angela Boškin Faculty of Health Care, Spodnji Plavž 3, 4270 Jesenice, Slovenija; bskelasavic@fzab.si (B.S.-S.); msmodis@fzab.si (M.S.); 8 Degree Course in Nursing, University of Modena and Reggio Emilia, Seat of Reggio Emilia, 42123 Reggio Emilia, Italy; sfinotto@unimore.it (S.F.); daniela.mecugni@unimore.it (D.M.)

**Keywords:** evidence-based practice, evidence-based nursing, undergraduate students, nursing students, nursing education, teaching EBP

## Abstract

*Background*: Evidence-based practice (EBP) is the appropriate approach to guide healthcare personnel in their clinical practice. Despite the importance of EBP, undergraduate nursing students are not very much engaged and have a lack of knowledge and skills. *Aim*: The aim of this study was to gather, assess and synthesize evidence on educational interventions promoting evidence-based practice competencies in traditional undergraduate nursing students. *Methods*: This is a scoping review on sixteen English and non-English databases. A data extraction form was established including authors, year of publication, country, types of participant, specific objectives, study design, educational intervention, comparison if existed, and outcomes of significance. *Results*: The search strategy retrieved 8901 records in total. After screening for duplicates and eligibility, 20 articles were included in the qualitative synthesis. Improvement in EBP domains such as knowledge, skills, attitudes/behaviours, EBP beliefs, use, practice, level of evidence, critical thinking and future use of EBP were mentioned and assessed in different studies. *Conclusions*: EBP training can improve nursing students’ capacity in healthcare provision. Teaching EBP competencies along undergraduate nursing curricula should be a high priority at nursing programmes. The use of innovative approaches seems to be more effective than traditional ways. Education of EBP increases its future use and critical thinking and EBP programs improve self-efficacy and the level of evidence utilization.

## 1. Introduction

Evidence based practice (EBP) is an approach that guides healthcare personnel decisions to use the best available research evidence with clinical expertise and patient’s unique values and preferences [1]. The main health professional organizations have set EBP as an important element of quality care, establishing EBP competency as a professional standard [2,3,4]. However, despite the fact that nurses think positively of EBP, they have a lack of knowledge and skills [5]. According to a study, nurses are less willing to support EBP promotion and implementation, and have less skills and knowledge in comparison to physicians [6].

Education level is strongly associated with EBP beliefs and its implementation, suggesting that university education increases the appreciation of this practice and instils the desire to use EBP also in clinical practice [7,8]. Although most faculties are supportive of teaching EBP, they do not incorporate EBP into their curricula due to high job demands or lack of skills and knowledge, and present practice has focused on the teaching research methodology [9,10].

Previous systematic reviews closely related to our topic have revealed that nursing education for EBP emphasizes information literacy as the most important competence for EBP [11]. Two scoping reviews pointed out that nursing educational strategies of evidence-based approaches vary with regards to content and implementation strategies and the most frequents are lectures, laboratory work, blended approaches, small classroom activities, educational sessions and EBP projects [12,13]. A very recent study reached the conclusion that EBP teaching differs in EU countries and in all cycles of education. Usually, it is part of other subjects and rarely is an autonomous course [14]. A thematic review of qualitative studies has shown that interactive teaching strategies are used alongside, however, collaboration with clinical practice was only vaguely addressed [15].

Nevertheless, international studies show a high variability at EBP competency of undergraduate nursing students [16,17]. The literature indicates that many nursing students are not very much engaged in EBP use and struggle to see the relevance of evidence for nursing practice due to lack of knowledge and skills, non-positive attitude towards EBP in students, faculties and nurses as well as the lack of support in clinical settings [11,12]. Embedding EBP in undergraduate nursing programs has been strongly recommended [18]. Earlier systematic reviews have been focused on the effectiveness of EBP teaching strategies for undergraduate health students [19,20,21,22], while other reviews focused on nursing students have exclusively focused on the effectiveness of EBP teaching methods to promote students’ critical thinking [23], without quantifying the effect of the strategies [12,13] or being oriented to qualitative studies [15].

Therefore, rigorous evaluation is needed to understand which educational strategies and innovations are most effective in preparing nursing students to enact EBP upon entering practice. The aim of this study was to gather, assess and synthesize evidence on educational interventions promoting evidence-based practice competencies in traditional undergraduate nursing students.

## 2. Methods

### 2.1. Design

We conducted a scoping review design which seeks to systematically search for, appraise and synthesis research evidence, in accordance with the PRISMA statement [24]. The review was carried out by an international partnership team that include 13 researchers from Czech Republic, Greece, Italy, Poland, Spain and Slovenia [25].

### 2.2. Eligibility Criteria

Based on the literature review, as eligible studies were those that met the criteria below:

1. Types of participants: undergraduate nursing students, incorporating professional or bachelor nursing programme degrees;
2. Intervention: Studies that focused on educational interventions aimed at improving EBP competency among nursing students;
3. Outcomes: Studies were included if they address at least one of the follow primary learning outcomes: participants’ attitudes, beliefs, knowledge, skills, self-efficacy, behaviour and competency on EBP. Secondary outcomes: reaction to the EBP educational experience, critical thinking and benefit to patients associated with EBP;
4. Study design: Studies that performed quantitative and qualitative estimates of the effectiveness of the EBP educational strategies were used, irrespective of the presence of comparator groups, including randomized controlled trial, quasi-experimental controlled trial and before and after studies, and no limit was placed on the duration of the follow-up;
5. Other criteria: Research papers that were published in peer-reviewed journals; published in English language and research team’s native languages (Czech, Greek, Italian, Polish, Spanish and Slovenian); from any geographical area. Studies that didn’t meet these criteria were excluded and studies that met the criteria were shortlisted for inclusion in the review. Additionally, some retrieved citations were excluded. They were the review articles, proceedings and dissertations.

### 2.3. Information Sources and Search Strategy

The research question that guided our search strategy was “What are the existing educational interventions used to promote evidence based practice among nursing students?” Derived from this, a protocol based on the PRISMA Guideline was developed and a scoping literature search from 2004 to July 2019 on sixteen English and non-English databases were conducted. The English databases reviewed were CINAHL, PsychInfo, Scopus, PubMed, EMBASE, Cochrane Library, Web of Science, ProQuest, EBSCOhost, Springer Link and ScienceDirect. As about the non-English databases they were: Spanish and Portuguese languages databases CUIDEN, IBECS, SciELO, LILACS, and Polish language database Polska Bibliografia Lekarska (PBL). The core algorithm used in the study was: ((“Students, Nursing” OR “Nursing” OR “Nurses”) AND (“Models, Educational” OR “Education” OR “Education, Distance” OR “Distance Learning” OR “Online Learning” OR “Online Education” OR “Health Education” OR “Education, Nursing, Graduate” OR “Teaching” OR “Curriculum” OR “Training” OR “Educative Innovations”)) AND (“Evidence-Based Practice” OR “Evidence-Based Nursing”). No language or other limitations were imposed (Table 1).

Besides this, we used manual searches, and two researchers reviewed the table of contents of journals that publish most of the studies related to this topic: *Nurse Education Today, Worldview on Evidence-based Nursing* and *Nurse Education in Practice*. Searches in reference lists of previously retrieved studies and literature reviews were also performed.

### 2.4. Study Selection

Two reviewers of each partner team independently screened 2–3 databases following the same protocol; as a result, each database was searched twice. Spanish team also searched in Spanish and Portuguese databases, and Polish team in its language database. A three stage approach was used in order to include/exclude studies in the final review process. Initially, duplicate studies were excluded and after that, screening process took place based on (1) title, (2) abstract and finally (3) the full text. Discrepancies regarding article selection were solved by consensus.

### 2.5. Data Collection Process, Extraction and Quality Assessment

The selected articles were reviewed by two independent reviewers. A data extraction form was established including authors, year of publication, country, types of participant, specific objectives, study design, educational intervention, comparison if existed, and outcomes of significance (mean and median differences of changes in attitude, knowledge, skills, behaviour or competency if available). The Critical Appraisal Skills Programme-CASP (https://casp-uk.net/casp-tools-checklists/) checklist for randomized controlled trial was used to evaluate the quality and bias risk of studies. Discrepancies between reviewers were discussed between the whole team. A quality assessment total score was calculated for each of the study finally included in the review: 11 was maximum possible score, meaning the highest quality. The studies that report educational intervention are known for their quality variability. Due to this, each study quality was evaluated independently and a risk of bias was pointed out for each.

### 2.6. Synthesis of the Results

The studies included in the final analysis had heterogeneity both for the intervention (type of educational intervention, content, duration and timing of the course) and the outcomes; few studies utilized a common, validated instrument to measure outcomes across knowledge, skills, attitudes and behaviour. An aggregative narrative synthesis of the included studies was performed. However, due to the high heterogeneity of the found studies, we decided to present the results based on the type of intervention (face-to-face, on-line or mixed).

## 3. Results

### 3.1. Literature Search

**Figure 1:**
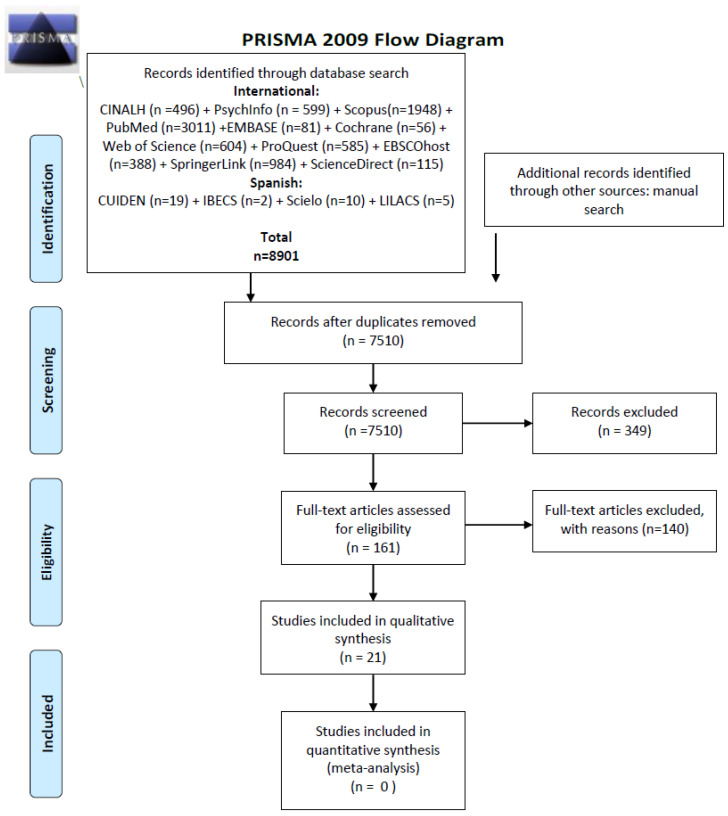
PRISMA 2009 Flow Diagram. From: Moher, D.; Liberati, A.; Tetzlaff, J.; Altman, D.G. The PRISMA Group Preferred Reporting Items for Systematic Reviews and Meta-Analyses: The PRISMA Statement. *PLoS Med*. **2009**, *6*, e1000097, doi:10.1371/journal.pmed1000097 [24]. For more information, visit www.prisma-statement.org.

Our search strategy retrieved 8901 records in total. After screening for duplicates and eligibility, 20 articles were included in qualitative synthesis. The whole process of study, identification and inclusion/exclusion is presented in Figure 1. The Prisma guidelines has been followed [24].

### 3.2. Studies Characteristics

Eight studies were conducted in USA, five studies were conducted in Europe, five in Asia, one in Australia and one in USA/Middle-East. Regarding their design, seventeen of them are “*quasi-experimental studies with pre-post-test*”, one used a mixed methods design [26], (quasi-experimental and two-population randomized controlled trial design), one is an *experimental study with one control group* [27] and the last is a *longitudinal panel study* [28].

The sample size varied from 32 to 292 students. Studies included on-line, face-to-face or mixed interventions. Some educational approaches provided credits and the courses/lectures/training lasted from thirty minutes to one semester. Other studies included EBP methods and techniques in the curricula. The studies included single (lectures/lessons/workshop/tutorials) or a combination of different approaches. Different aspects of EBP were included during the interventions such as introductory lessons, collaborative learning techniques, quality assessment of literature, critical appraisal of the literature, combination of research methodology, process and ethics, research methods and research utilization skills, searching strategies, work in small groups and discussion and incorporation of EBP in clinical practice.

Ten studies used valid instruments to assess the EBP knowledge, attitudes and beliefs. Among the studies, three of them used the EBP-COQ questionnaire [29,30,31], one the S-EBPQ tool [32], one the EBASE survey [33] and one the KAB questionnaire [34]. Two studies were focused on research and, more specifically, one on attitudes toward research and one on confidence in research and EBP belief [26,35]. All but three studies were focused on cognitive issues (cognitive learning outcomes, cognitive load scale and cognitive knowledge related to EBP). Additionally, in two studies, among other things, the EBP implementation scale was assessed [28,36]. A specific focus was given on EBP beliefs in three studies [28,36,37]. In these studies, the authors used a reliable and validate EBP belief scale.

The CASP score ranged from 1 to 9. One study had a CASP score of nine [29], four studies had a score of four [28,30,35,38], two studies [36,39] had a score of one and four studies a CASP score of three [26,40,41,42]. Additionally, by two studies had a score of five [34,43], six [32,44] and seven [31,45].

### 3.3. Data Synthesis

Four studies showed an overall statistical significant increase in EBP domains such as knowledge, skills and attitudes/behaviours [29,30,31,34] while other studies show a significant increase only on the knowledge level [38] whilst other studies show an increase, but it was not statistically significant [32]. Significant improvement in EBP competences was observed on three studies [29,30,31]. However, three studies report a significant improvement in EBP beliefs [28,36,37]. Improvement in other significant indicators such as EBP use [33], EBP practice [36], self-efficacy and level of evidence utilization [43], critical thinking and future use of EBP [29] were mentioned and assessed in different studies.

Eleven studies included in the review used a face-to-face intervention (Table 2). The most effective interventions were those which last for around one semester [27,31,35]. The use of different approaches such as lecturers, tasks, discussion in class, individual work and use of small groups increase the effectiveness of an EBP program. Additionally, the use of interactive methods (i.e., use of PowerPoint) is an added value for an effective EBP program [27]. However, a combination of lectures and practice was found to be effective [29]. One study focused on assessment of the effectiveness of educational programs on undergraduate nursing students that provide them knowledge, skills and promote a positive attitude toward EBP [34]. The results indicate a significant increase in EBP knowledge (11.51 vs. 17.11; *p* < 0.001), EBP attitudes and beliefs (35.67 vs. 38.99; *p* < 0.001) and EBP behaviour (10.99 vs. 15.32; *p* < 0.001). In a Spanish study among second- and third-year nursing students, a 15-week educational intervention (60 class hours and 90 h of student work) took place [31]. Participants of the intervention group reported a statistically significant (all at a level of *p* < 0.001) improvement in EBP knowledge (2.82 vs. 3.92), skills (2.75 vs. 4.01), attitudes (3.33 vs. 4.28) and competences (3.06 vs. 4.11) while the change in the control group was small. A study conducted in the USA assessed the effectiveness of an interactive teaching strategy on EBP [38]. After the intervention, the experimental group mean score was higher for EBP knowledge (5.15 vs. 5.68; *p* = 0.001), EBP use (2.27 vs. 2.62; *p* = 0.015), attitudes toward EBP (4.69 vs. 4.78; *p* = 0.398) and future use of EBP (4.81 vs. 5.17; *p* = 0.255). Authors reached the conclusion that this strategy could improve both knowledge and EBP use among nursing students but not their attitudes or the future EBP use. A study that evaluated the effectiveness of an EBP program, found that immediately after the program, the intervention group had statistically more significant scores than the control group on competencies (3.04 vs. 4.34), future use of EBP (4.01 vs. 4.33), EBP knowledge (3.34 vs. 4.27), EBP attitudes (3.77 vs. 4.33) and critical thinking (3.29 vs. 3.72) [29]. At a follow up six weeks after the program, the experimental group had higher scores in comparison to control group in EBP knowledge (3.33 vs. 4.16), EBP skills (3.65 vs. 7.66), EBP attitudes (3.72 vs. 4.24), EBP competencies (3.14 vs. 4.18), future use of EBP (3.84 vs. 4.38) and critical thinking (3.30 vs. 3.78).

However, the findings of another study that aimed to compare the impact of embedding EBP in the nursing curricula with modular-based teaching are not statistically significant [32]. The differences found between the modular and embedded curriculum groups were not significant as regards the EBP implementation, EBP attitudes, knowledge and skills in retrieving and reviewing evidence and knowledge and skills in applying and sharing EBP. Other study evaluated the effectiveness of EBP as a pillar program [28]. To measure EBP beliefs and EBP implementation scale, the Melynk’s self-report questionnaire was used. Statistical significant results were found before and after the course at the EBP belief scale (3.89 vs. 4.25; *p* < 0.001) and at the EBP implementation scale (2.68 vs. 3.61; *p* < 0.001). Similar results are also reported by another study [37]. The EBP Belief scale was significantly improved after the eight half-day workshops provided to undergraduate nursing students (59.0 vs. 66.4). After a follow-up period from 1 to 25 months, for nurses’ preceptors, an increase in EBP of 52% was reported. Another study found that students’ perceptions and confidence in research can be improved by an effective research and EBP course, with most of the students agreeing that they plan to use EBP skills in the future [35]. An experimental study with one control group evaluated the effectiveness of integrating constructivist theories and EBP on student cognitive load and learning performance in a research course [27]. The control group had lower mental efficiency (0.31 vs. 0.33), mental effort (10.07 vs. 11.07), mental load (7.27 vs. 8.74) and research knowledge (44.92 vs. 70.61) in comparison to the intervention group. A journal club was embedded in a nursing research course and the effect on students learning was assessed [40]. At the start of the semester, a questionnaire with 14 questions was completed by participants and at the end of the semester (post-journal club), four more questions were added to the initial questionnaire. A total of 70% of the participants made positive statements about their participation at the journal club or declared that it was helpful for learning how to critically appraise research. A quasi-experimental study was conducted in Taiwan on 2nd year nursing students [44]. Authors developed innovative teaching strategies to improve nurses’ involvement in research. After the intervention, the experimental groups had higher scores on attitudes toward research (65.8 vs. 73.0), classroom engagement (34.8 vs. 37.3), nursing competences scale (28.9 vs. 33.5) and other key dimensions in comparison to the control group.

Four studies included in the review used an on-line intervention approach (Table 3). The duration of the intervention ranged from 30 min to 16 weeks. The use of a 30-min video (through a web-based application) can improve research skills and appraisal skills, while the use of virtual simulation is effective in EBP [26,41]. However, the combination of discussions, tasks, assignments, exams and preparation of a project have been shown to be effective in practicing EBP by nursing students [42]. Authors examined the effectiveness of an on-line research/EBP course [42]. Despite the fact that the overall EBP mean total scale scores improved significantly after the EBP course (86.11 vs. 105.69; *p* = 0.001), differences in knowledge (23.43 vs. 25.23; *p* = 0.163) and attitudes (20.69 vs. 21.11; *p* = 0.433) toward EBP were not found. A study conducted in the USA and Middle East assessed the effectiveness of the Evidence-Based Research Tool in improving on-line research and critical appraisal skills [26]. Authors’ findings report that the research skills of students are significantly enhanced by the use of EBPR. Another study performed a 30-min virtual simulation exercise by using the Internet-based platform of CliniSpace and followed with a post-test [41]. A self-reported questionnaire was used to evaluate cognitive knowledge related to EBP and affective knowledge about how evidence affects clinical decision-making. There was a statistical significance difference in cognitive knowledge related to EBP (60% vs. 80%; *p* < 0.0001) before and after the intervention.

Five studies used a blended approach in provision EBP training to nursing students (Table 4). The duration ranged from 4 h (2 h of theory and 2 h of computer lab sessions) to one academic year [30,36]. A combination of lectures, voice-over PowerPoint, videos, team-based learning, computer-based learning, small groups, individual projects and problem-based learning have been shown to be effective in different studies [30,36,43,45]. A study evaluated the effectiveness of an educational intervention (two hours of EBP theory and two hours of computer lab sessions) with pre- and two post-measures. The findings showed statistically significant differences in attitude (3.47 vs. 3.55) and knowledge (2.82 vs. 3.36) of the students, but not in the EBP skills (2.94 vs. 2.92), two months later [30]. A second measurement at the end of the semester (12-weeks after the start of the semester) did not show a statistical difference between the intermediate and final measurement (*p* = 0.092). The mean score for attitude, knowledge and skills at the final measurement was 3.55, 3.45 and 3.02, respectively [30]. A 16-week research educational program conducted in Australia among undergraduate nursing students reached the conclusion that this kind of program could significantly improve the score on EBP skills (38 vs. 43; *p* < 0.001) and EBP use (5 vs. 7; *p* = 0.002), but not median EBP attitude sub-score (33 vs. 33; *p* = 0.280) [33]. A study in the United Kingdom assessed the attitudes, beliefs, knowledge level and utilization of EBP among undergraduate nursing students [36]. The results emphasize the statistically significant change in EBP belief scale (in seven among sixteen categories) and on the EBP implementation scale (in thirteen among eighteen categories). In another study, after provision of an intensive EBP education program for 30 h, EBP self-efficacy was statistically (*p* < 0.001) improved at the intervention group in comparison to the control group [43].

## 4. Discussion

The use of EBP in nursing students’ education is of paramount significance for their future clinical practice. The educational interventions that exist for nursing students vary in duration, methods provided, and if the approach is single or combined. Despite the heterogeneity of EBP educational intervention programs, we can say that the EBP educational approaches can increase knowledge, skills and competency as well as improve the beliefs, attitudes and behaviours of nursing students. It is very important to get this basic professional frame on undergraduate education programs because translation of all these into daily practice can significantly improve the clinical outcome. According to the current study, face-to-face approaches seem to be more effective in comparison to on-line or mixed interventions. The effect size of the face-to-face interventions was higher in comparison to the other methods. However, these results should be interpreted with caution as the number of studies that used on-line or a mixed approach is low and follow-up was carried out in very few studies. Even in these studies, the follow–up was not very longitudinal.

According to the results of the current studies, EBP education approaches should be blended. A combination of lectures, discussions, exams, assignments, small group works, team-based learning and individual learning are shown to be effective in different studies. Despite the large use of technology and web-based applications/apps, the results about their effectiveness in teaching EBP are still limited. Regarding the duration of the EBP program, most of the studies conclude that one semester is very effective. However, even EBP programs that did not last as long showed effectiveness.

Nursing student education in EBP programs increases the future use of EBP and critical thinking [29,38]. Additionally, the students’ mental load, mental effort, and mental efficiency can be increased by the integration of EBP in research programs [27]. The EBP programs are of paramount significance for the research skills of students as well as for their confidence in research [26,35]. Their skills can be improved and used for future research. Our findings also indicate the improvement in self-efficacy and the level of evidence utilization by the EBP programs [43]. Similar results of undergraduate students are shown in a study which concluded that EBP training improves the knowledge and critical appraisal skills of dental students [52]. Additionally, training in EBM practice increases the knowledge and self-perception of competencies in medical students [53].

The EBP programs are provided in different academic years for nursing students and drawing a conclusion about the most appropriate study year is difficult. Additionally, huge differences exist between countries in nursing education and their future role in the provision of healthcare services. In some countries, the bachelor’s degree last three years while in others it lasts four. Additionally, the number of ECTCs differs between countries. The educational teaching and learning strategies for EBP in nursing education need to use innovative and interactive approaches. Similar results are presented to another literature review where finding innovative ways is a huge challenge for nursing academics [19]. The innovative approaches increase student interest and enthusiasm. The learning strategies that have enhanced EBP aspects in nursing education effectively prepare the new healthcare personnel to deal with challenges in their future daily practice.

Our findings emphasize the importance of including EBP training in the curricula of nursing schools. The introduction of EBP in nursing curricula was implemented after students’ positive feedback [54] on a video EBP project, while other studies provide information on designing evidence-based practice curricula [55]. However, this needs a strong leadership and a shift in institutions’ culture. The development of EBP educational programs is strongly recommended by another study [20].

To the best of our knowledge, this is among the first studies to examine such an important topic including articles not only in English but in five more languages. Additionally, we searched different electronic databases, making this representative of the nursing student population. The use of the CASP tool in the analysis is a strength of the current study. One of the limitations is the heterogeneity of the studies included in the analysis, something which made a meta-analysis not possible. Additionally, we included only undergraduate nursing students and the results of this study are not representative for graduate or master’s students.

## 5. Conclusions

To sum up, EBP training can improve nursing students’ capacity in healthcare provision. The provision of EBP programs is recommended to be a combination of lectures, seminars, discussions, exams, assignments, small group works and project works, team-based learning, case-study analysis and individual learning. To date, the most effective teaching method seems to be the face-to-face. Teaching EBP competencies by using interactive approaches along undergraduate nursing curricula should be high priorities for nursing programmes. The EBP programmes’ duration can be one semester and they can be part of the normal education procedure of the nursing students. EBP training needs to start at the undergraduate level and develop competences at the master’s and PhD levels. Further work is required to assess the most effective way as well as the duration of delivering EBP training to the nursing students.

## Figures and Tables

**Table 1:** Search strategy used for the study.

Mesh Terms	
Nursing	1. Students, Nursing
	2. Nursing
	3. Nurses
	**1 or 2 or 3**
Teaching strategies-educative innovations	4. Models, Educational
	5. Education
	6. Education, Distance
	7. Distance Learning
	8. Online Learning
	9. Online Education
	10. Health Education
	11. Education, Nursing, Graduate
	12. Teaching
	13. Curriculum
	14. Training
	15. Educative Innovations
	16. Blended learning
	**4 or 5 or 6 or 7 or 8 or 9 or 10 or 11 or 12 or 13 or 14 or 15 or 16**
Evidence-Based Practice—EBP	17. Evidence-Based Practice
	18. Evidence-Based Nursing
	**17 or 18**

**Table 2:** Data extraction for face to face used method to teach EBP.

Author et al. (Year)	Main Study Characteristics (Country, Years of Study, Population, Sample Size)	Aim of the Study	Type of Study	Intervention (Delivery Option, Type, Duration etc.)	Instrument (Name, Structure, Scale etc.)	Main Findings	CASP Score
Kim et al. (2009) [38]	USA, 2007–2008 Nursing students. The experimental group (EG) (*n* = 88) received the E-FIT strategy intervention and the control group (CG) (*n* = 120) received standard teaching.	To assess the effectiveness of the evidence-based practice (EBP)-focused interactive teaching (E-FIT) strategy	Quasi-experimental, pre- and post-test study design with a control group	**E-FIT intervention** included a 2-h introductory lesson on the basic EBP principles and processes, as well as a description of the clinically integrated EBP group projects to be carried out throughout the semester in partnership with clinical preceptors at respective healthcare facilities.	Knowledge, Attitudes and Behaviors Questionnaire for EBP (Johnston et al. 2003) [46]	Post-test: **EBP Knowledge** (mean difference = 0.25; *p* = 0.001) **EBP Use** (mean difference = 0.26; *p* = 0.015) **Attitudes toward EBP** mean difference = −0.12; *p* = 0.398 **Future Use of EBP** mean difference = 0.13; *p* = 0.255	4
Zhang et al. (2012) [34]	China, 2009–2010 Undergraduate Nursing students (*n* = 74)	To evaluate the effectiveness of an educational program on the knowledge, attitudes and beliefs, and behaviour of EBP in undergraduate nursing students as well as to promote independent learning and cooperative abilities through SDL and workshop learning strategies.	Quasi-experimental, pre- and post-test study design without a control group	During clinical practice period in the last year: **Phase 1 Self-Directed Learning (SDL) Process for EBP:** Each learning package consisted of an introduction letter about learning process and requirements, a booklet for learning resources and objectives, and journal articles for workshop use. **Phase 2 Workshop for Critical Appraisal of Literature:**	**Self-report questionnaire:** KAB, contained 30 items which can be subdivided into three dimensions, that is, knowledge, attitudes and beliefs, and behavior about EBP. Last part asked questions about participants’ subjective view and satisfaction of their learning process. One open-ended question to assess their evaluation	**Knowledge EBP ranging 8 to 24:** Pre-test 11.51 ± 2.51 Post-test 17.11 ± 3.30 *p* < 0.0001 **Attitudes EBP ranging 14 to 42:** Pre-test 35.67 ± 5.43 Post-test 38.99 ± 4.04 *p* < 0.0001 **Behaviour EBP ranging 8 to 24:** Pre-test 10.99 ± 2.77 Post-test 15.32 ± 3.14	5
Liou et al. (2013) [44]	Taiwan, 2010, nursing students who were enrolled in a 2-year Registered Nurse-to-Bachelor of Science EG = 103 CG = 106	To develop innovative teaching strategies that increase nursing students’ interests and engagement in research	Quasi-experimental, pre- and post-test study design with a control group	Intervention: innovative teaching strategies for a research course: collaborative learning techniques and evidence-based practice of learning were expected	-Attitudes toward Research Questionnaire; -Classroom Engagement Scale; -Self-Directed Learning Instrument; -Nursing Eight Core Competencies Scale; -Value of Teams survey; -Research knowledge test (objective test) A 10-item multiple choice test	Research knowledge test: **Attitudes toward Research:** Control: Pre-Test 64.7 (SD = 6.10) Post-test 65.8 (SD = 8.88) Experimental: Pre-test 64.5 (SD = 6.69) Post-test 73.0 (SD = 6.53) **Classroom Engagement Scale:** Control: Pre-test 34.4 (SD = 3.11) Post-test 34.8 (SD = 4.25) Experimental: Pre-test 35.5 (SD = 3.41) Post-test 37.3 (SD = 3.86) **Value of Teams:** Control: Pre-test 63.4 (SD = 4.45) Post-test 62.8 (SD = 6.07) Experimental: Pre-test 63.6 (SD = 4.64) 65.8 Post-test (SD = 4.43) **Self-directed Learning Instrument:** Control: Pre-test 74.5 (SD = 6.84) Post-test 76.0 (SD = 8.14) Experimental: Pre-test 75.5 (SD = 6.25) 78.9 Post-test (SD = 6.64) **Nursing Eight Core Competencies Scale:** Control: Pre-test 30.1 (SD = 3.31) Post-test 28.9 (SD = 4.37) Experimental: Pre-test 30.9 (SD = 3.44) Post-test 33.5 (SD = 3.56) Experimental group participants had a lower degree of pressure, and higher degrees of interest, enjoyment, and acceptance of the research course than the control group participants.	6
Ruzafa-Martínez et al. (2016) [31]	Spain, 2010 Second-year nursing students (Bachelor Degree) (EG: *n* = 61) (CG: *n* = 59)	To evaluate the effectiveness of an EBP course on the EBP competence undergraduate nursing students	Quasi-experimental, pre- and post-test study design with a control group	**Intervention:** The 15-week educational intervention, it comprised 60 h in class plus 90 h of student work, with a minimum attendance requirement of 80%. Focused on the first four steps of EBP described by Melnyk et al.	Self-report questionnaire: **EBP-COQ**, a validated Spanish-language instrument specifically developed to evaluate the self-perceived EBP competence level in nursing students (Ruzafa-Martinez et al., 2013) [47]. Domains: Attitude, Knowledge and Skills.	**EBP Competence (score 1–5):** CG: mean(95%CI) Pre: 3.37 (3.25–3.50) Post: 3.62 (3.51–3.73) EG: mean (95%CI) Pre: 3.06 (2.93–3.19) Post: 4.11 (4.01–4.22) ANOVA time × group: *p* < 0.001 **EBP Attitude (score 1–5):** CG: mean (95% CI) Pre: 3.84 (3.65–4.03) Post: 3.92 (3.80–4.05) EG: mean(95%CI) Pre: 3.33 (3.14–3.52) Post: 4.28 (4.16–4.41) ANOVA time × group: *p* < 0.001 **EBP Knowledge (score 1–5):** CG: mean(95%CI) Pre: 2.51 (2.32–2.71) Post: 3.01 (2.87–3.15) EG: mean(95%CI) Pre: 2.82 (2.62–3.02) Post: 3.92 (3.77–4.06) 4.01ç ANOVA time × group: *p* < 0.001 **EBP Skills (score 1–5):** CG: mean(95%CI) Pre: 3.20 (3.01–3.38) Post: 3.49 (3.32–3.65) EG: mean(95%CI) Pre: 2.75 (2.56–2.94) Post: 4.01 (3.85–4.18) ANOVA time × group: *p* < 0.001	7
Hsieh, et al.(2016) [27]	Taiwan, 2012–2013, Registered Nurse-to-Bachelor of Science in Nursing program. Control group (*n* = 97) underwent “traditional lecture-based research course” Experimental group (*n* = 90) experienced “EBP-based research course” Randomly allocated by class	To examine the effect of integrating constructivist and evidence-based practice on student cognitive load and learning performance in a research course.	Experimental study with one control group.	Both group had three hours of instruction every week for thirteen weeks **Intervention:** EBP, cognitive theory, and constructivism were used to design the educational program. EBP was integrated into the research course with PowerPoint slides, the Great Cookie Experiment, exemplar analyses, asking questions and discussion, peer instruction, group discussion, individualized team guidance, research article critique and practice. **Control:** The standard research program included reading a required textbook, attending didactic lectures with PowerPoint slides, asking questions, and participating in group discussions.	Self-report Questionnaires: Cognitive Load Scale (CLS) Objective test: -Research Cognitive Test (0–100) includes 20 item multiple choice questions and 3-item essay questions based on the teaching objectives of each unit, to measure students’ individualized learning performance. -Mental efficiency -Team paper critique (0–100) -Qualitative feedback on course satisfaction	The mental load, mental effort, mental efficiency and research knowledge were higher in the research group than in the control 8.74 vs. 7.27, *p* < *0*.001; 11.07 vs. 10.07, *p* = 0.009; 0.33 vs. −0.31, *p* < 0.001 and 70.61 vs. 44.92, *p* < 0.001 respectively. Additionally, introduction literature review and assignment requirement and writing were higher in experimental group than the control.	8
Keib, et al. (2017) [35]	USA, 2012–2013 Third-year BSN students (2012, *n* = 55) (2013, *n* = 54)	The changes in perceptions, confidence and interest in research and EBP among nursing students were assessed.	Quasi-experimental, pre- and post-test study design without a control group	Intervention: **Research and EBP course** 3-credit hour, semester-long course introduced students to research and EBP concepts and required the completion of an EBP project that was presented at the conclusion of the course in an interprofessional poster session with nursing and pharmacy faculty and students. Methodology: included lectures, seminar assignments and discussions, and small group work.	**Self-report questionnaire:** Confidence in Research and EBP survey. Created by the investigators. The Research and EBP Perceptions items assessed students’ perceptions of the importance and usefulness of research, their understanding of research and EBP, and their plans to utilize research and EBP in their future practice.	Results are showed item by items, no global score. **Research perceptions:** **In 2012:** Understood difference between research and EBP (*p* < 0.001), research and EBP complement each other (*p* < 0.001), the usefulness of research in the nursing profession (*p* = 0.003). They more highly rated the amount of research experience they have in comparison to their peers (*p* = 0.021). More students strongly agreed they planned to use EBP in the future (*p* = 0.007). **In 2013:** More students agreed they planned to use EBP in the future (*p* = 0.007). **Confidence in Research items** In 2012 and 2013, there were significant improvements in all 19 Confidence in Research items	4
Scurlock-Evans et al. (2017) [32]	UK, 2011–2014 pre-registration nursing students (*n* = 56)	To compare the impact of embedding EBP throughout the curriculum, with modular-based teaching, on pre-registration nursing students’ EBP profiles.	A longitudinal panel study design.	**Modular-curriculum:** research methods and research utilization skills through the same research methods module (in year 2 of their studies) **Embedded-curriculum:** All students are taught how to assess quality of literature/evidence in year 1; what EBP is, how it links with research methodology and process and ethics in year 2, and; all students then undertook an independent research project in their final year (which was explicitly packaged as cementing their research methods and EBP skills).	Self-report questionnaire: **Student Evidence-Based Practice Questionnaire (S-EBPQ)** completed in the first, second and third year of their course.	**EBP implementation:** differences overall between the modular- and embedded- curriculum groups (F(1, 54) = 1.11, *p* = 0.237; No statistically significant interaction effect between time and curriculum type (F(2, 108) = 3.67, *p* = 0.029 **Attitudes towards EBP:** no statistically significant differences between the two curriculum-type groups overall (F(1, 54) = 2.31, *p* = 0.135,) and no significant interaction between time and curriculum-type (F(1.71, 92.05) = 0.79 **Knowledge and skills in retrieving and reviewing evidence:** no significant difference overall between the two curriculum-type groups (F(1, 54) = 0.96, *p* = 0.331), a statistically significant interaction between time and curriculum-type was identified with a large sized effect (F(2, 108) = 6.34, *p* = 0.002 **Knowledge and skills in applying and sharing EBP:** no significant difference overall between the two curriculum-type groups (F(1, 54) = 0.02, *p* = 0.888), a statistically significant interaction between time and curriculum-type was identified with a large-sized effect (F(2, 108) = 7.14, *p* = 0.001	6
Singleton (2017) [28]	USA, 2008–2015, Doctor of Nursing Practice-Family Nurse Practitioner students (*n* = 54)	To assess the impact of a curriculum on EBP beliefs and implementation in Doctor or Nursing Practice-Family Nurse Practitioner (DNP-FNP) students.	Quasi-experimental, pre- and post-test study design without a control group	**EBP curriculum** EBP was identified as a program pillar and was threaded across all courses. The curriculum also included two specific EBP methods and technique courses, and three DNP project courses. During the DNP project courses, students worked as teams with a faculty mentor and a mentor from a clinical partner agency to complete an evidence-based clinical practice improvement project for the clinical partner agency. Faculty EBP mentors, all of whom were actively engaged in EBP, were embedded across the courses. The intensity of faculty mentorship increased as students began their DNP project work.	**Melnyk´s Self-report questionnaires:** -EBP Beliefs Scale (EBP-B) -EBP Implementation Scale (EBP-I)	EBP Beliefs Scale (EBP-B) pre-measure mean score was 3.89, and post-measure was 4.25. *p* < 0.001 EBP Implementation Scale (EBP-I)f: pre-measure mean score was 2.68, and post-measure was 3.61. *p* < 0.001	4
Hagler et al., (2012) [37]	USA, 2009–2011 Nurse preceptor participants recruited from seven hospitals (*n* = 160)	To test the effectiveness of a workshop designed to increase preceptor knowledge and endorsement of evidence-based practice	Quasi-experimental, pre- and post-test study design without a control group	A workshop for clinical experiences was held for eight and half days.	A questionnaire was completed before the workshop and was consisted of 21 questions related to demographic characteristics and 16 to EBP Beliefs Scale. The EBP scale was completed also after the workshop.	The EBP Beliefs Scale was improved significantly from pre-test to post-test (Mpre = 59.0, SDpre = 8.4, Mpost = 66.4, SDpost = 6.8, *p* < 0.001. The use of EBP was increased at a follow up period (1 to 25 months).	8
Kim et al. (2019) [29]	Republic of Korea, Fourth-year nursing students, Bachelor in Nursing (EG: 22, CG: 22) MaRtA010711	An education program evaluated the knowledge, skills, attitudes, competencies, and future use of EBP.	Quasi-experimental, pre- and post-test study design with a control group (pre-2 post; post1 2 weeks, post2 6 weeks)	**Intervention:** A 5 step EPB program of 4 weeks (8 sessions/20 h) took place. The program was based on Sackett et al. On the control group, no intervention was done.	Self-report questionnaire: Different instruments were used to measure (1) **Knowledge of EBP and attitudes toward EBP** (EBP COQ), (2) **Competencies for EBP** (12 items measured on a 5-point Likert scale; “EBP Competencies”, Essential Competencies for Evidence-Based Practice in Nursing), (3) **Future Use of EBP** (7 items measured on a 5-point Likert scale) of the Knowledge, Attitude and Behavior Questionnaire (KABQ), (4) **Critical Thinking** (35 items measured on a 5-point Likert scale Critical Thinking Disposition Scale) and (5) **EBP Skills** (Cognitive Skills of Evidence-Based Practice).	**EBP knowledge** (mean, SD) EG: Pre: 3.26 ± 0.40; Post1: 4.27 ± 0.51; Post2: 4.16 ± 0.46 CG: Pre: 3.27 ± 0.46; Post1: 3.34 ± 0.55; Post2: 3.33 ± 0.67 Group × time: *p* < 0.001 **Competences EBP** (mean, SD) EG: Pre: 3.06 ± 0.48; Post1: 4.34 ± 0.37 Post2: 4.18 ± 0.36 CG: Pre: 3.04 ± 0.61; Post1: 3.04 ± 0.51 Post2: 3.14 ± 0.55 Group × time: *p* < 0.001 **EBP Attitude** (mean, SD) EG: Pre: 3.67 ± 0.36; Post1: 4.33 ± 0.42 Post2: 4.24 ± 0.42 CG: Pre: 3.79 ± 0.32; Post1: 3.77 ± 0.34 Post2: 3.72 ± 0.34 Group × time: *p* < 0.001 **Future use of EBP** (mean, SD) EG: Pre: 3.83 ± 0.51; Post1: 4.33 ± 0.44 Post2: 4.38 ± 0.47 CG: Pre: 3.84 ± 0.55; Post1: 4.01 ± 0.56 Post2: 3.85 ± 0.46 Group × time: *p* < 0.001 **Critical thinking** (mean, SD) EG: Pre: 3.39 ± 0.35; Post1: 3.72 ± 0.36 Post2: 3.78 ± 0.37 CG: Pre: 3.35 ± 0.49; Post1: 3.29 ± 0.42 Post2: 3.30 ± 0.47 Group × time: *p* < 0.001 **EBP Skills** (mean, SD) EG: Post1:9.01 ± 1.52 CG: Post1: 3.44 ± 1.45; *p* < 0.001 EG: Post2: 7.66 ± 1.62; CG: Post2: 3.65 ± 1.63; *p* < 0.001	9
Diaz and Walsh (2018) (HAND-SEARCHING) [40]	USA, 32 students in their third year of study	To determine what effect a journal club as part of a nursing research course has on student learning in the undergraduate nursing classroom.	Quasi-experimental, pre- and post-test study design without a control group	A Journal club was incorporated at the curriculum with different meetings taking place after the regular classes. A meeting was held before the start of the semester and one at the end of the semester.	At the beginning a questionnaire with 14 questions was used while at the end a questionnaire with 18 items was used.	The 70% of participant reported positive statements about being part of the Journal club or found it helpful for learning how to critically appraise research.	3

Acronyms: Evidence based practice (EBP); focused interactive teaching—E-FIT; Knowledge, Attitudes and Behavior—KAB; Team-based learning—TBL; Evidence-based research—EBR.

**Table 3:** Data extraction for on-line/by distance used method to teach EBP.

Author et al. (Year)	Main Study Characteristics (Country, Years of Study, Population, Sample Size)	Aim of the Study	Type of Study	Intervention (Delivery Option, Type, Duration etc.)	Instrument (Name, Structure, Scale etc.)	Main Findings	CASP Score
Justham and Timmons (2005) [39]	UK, 2002, post-registration nursing students (*n* = 292)	The learning and attitude of post-registration nursing students were assessed after a web-based statistics test to teach statistics	Quasi-experimental. Pre-post-test study without control group	A 20 credits module titled ‘Evidence-Based Practice’ where an online statistics test was administered via WebCT site. The module addresses issues concerning the critical analysis and evaluation of a variety of sources of evidence relevant to patient/client care	Learning: statistic test from the WebCT test (objective test). Attitude: ‘Evaluation of the WebCT’ questionnaire by post (21 items)	Learning: First (pre) practice test mean percentage score 52.1%, and Second (post) assessed test was 92.6% (*p* = 0.000) Attitude: According qualitative data students have positive views with the use of the WebCT.	1
Long et al. (2016) [26]	USA. and Middle-East (ME) 2013–2014 Quasi-experimental nursing students enrolled in the introductory courses in US, RN-BSN and MSN and in ME BSN (*n* = 23) RCTs undergraduate nutrition, and PharmD students (*n* = 159)	To report the results of the effectiveness of the EBR tool to improve the overall online research and critical appraisal skills of learners engaged in EBP.	Mixed-method, quasi-experimental, and two-population randomized controlled trial (RCT) design	Intervention: **EBR tool** (*interactive technology-based tool usable from a computer, smartphone, or iPad to support student acquisition of online research and critical appraisal skills needed for EBP*) Subjects received the same 30-min video training, standardizing the study protocol and explaining how to access and use the tool. The video protocol requested participants to work through all ten steps, open and explore every hyperlink, and answer the embedded questions to help guide the online literature search assignment. A library link specific to each institution was placed within the EBR tool, allowing participants to access their institution’s library.	**Self-report Research questions:** The EBR tool pre- and post-test, does the EBR tool intervention (a) improve the overall research skill of users? Does it (b) improve the ability of the user to distinguish the credibility of online source materials?	Quasi-experimental results (nursing students). **Research skills:** **US/BSN:** T1 (M) = 3.50 (SD 0.70) T2 (M) = 2.88 (SD 0.98) d = 0.62 (SD 0.81) (CI 95% 0.40–0.83); *p* = 0.001 **ME/BSN:** T1 (M) = 3.27 (SD 0.93) T2 (M) = 2.32 (SD 0.64) d = 0.95 (SD 0.84) (CI 95% 0.58–1.32); *p* = 0.001 **Ability to distinguish credibility of online sources:** US/BSN (58), *p* = 0.057, ME/BSN (22), *p* = 0.219, US/MSN (41), *p* = 0.070.	3
Foronda et al. (2017) [41]	USA., 2016, master’s entry-level nursing students (*n* = 51)	To examine the impact of an in-class, group virtual simulation exercise on nursing students’ (a) cognitive knowledge of EBP and (b) affective knowledge about how evidence affects clinical decision-making.	Quasi-experimental, pre- and post-test study design without a control group	Virtual simulation using the Internet-based platform of CliniSpace and followed with a post-test. The entire exercise lasted about 30 min.	**Self-report questions:** Cognitive knowledge related to EBP five multiple-choice questions were asked. Affective knowledge about how evidence affects clinical decision-making.	**Cognitive knowledge related to EBP:** Median pretest 60% (IQR = 20) Median post-test score was 80% (IQR = 20) (*p* < 0.0001) **Affective knowledge about how evidence affects clinical decision-making** Pretest: 35% (agree) and 63% (strongly agree). Post-test scores increased to 23% rating it a 4 (agree) and 77% (strongly agree). **Not *p* value shows**	3
Rojjanasrirat and Rice (2017) [42]	USA. 2011–2012 Nursing master students (*n* = 63)	To examine whether or not EBP content provided early in an online, graduate research/EBP course would change the knowledge, attitudes, and practice of EBP of students from before to after taking the course.	Quasi-experimental, pre- and post-test study design without a control group	**Intervention:** Online research/EBP course This 4-credit hour graduate nursing course was taught across a 16-week trimester in the first semester of the MSN curriculum. On-line classroom strategies were used to teach research content and the EBP process including weekly asynchronous discussions, case study analysis, quizzes/exams, research critique, and a final EBP project that incorporated all elements of the EBP process	Self-report questionnaire: **Evidence-Based Practice** **Questionnaire (EBPQ)** (Validated by Upton, 2006) [48] Skill/Knowledge, Attitude, Practice **Facilitators and barriers to learning EBP concepts** developed by the principal investigator were also included, consisted of 14 structured items	**Overall EBPQ** mean scores significantly improved after taking the EBP course (t (63) = −9.034, *p* < 0.001). **Practice of EBP** Post-test: (M = 74.06, SD = 9.04) Pre-test scores (M = 55.29, SD = 11.57, t (63) = −12.78, *p* = 0.001). **Knowledge of EBP** Post-test (M = 25.23, SD = 9.9) Pre-test (M = 23.42, SD = 9.41; *p* = 0.79 **Attitudes toward EBP** Post-test: (M = 21.11, SD = 3.51) Pre-test (M = 20.69, SD = 3.51); *p* = 0.43	3

Acronyms: Evidence based practice (EBP); focused interactive teaching—E-FIT; Knowledge, Attitudes and Behavior—KAB; Team-based learning—TBL; Evidence-based research—EBR.

**Table 4:** Data extraction for both face to face and on-line/by distance used method to teach EBP.

Author et al. (Year)	Main Study Characteristics (Country, Years of Study, Population, Sample Size)	Aim of the Study	Type of Study	Intervention (Delivery Option, Type, Duration etc.)	Instrument (Name, Structure, Scale etc.)	Main Findings	CASP Score
Whittaker (2015) [45]	USA. 2012–2013, Students enrolled in a second degree baccalaureate nursing program or a Registered Nurse completion program. CG (*n* = 98) EG (*n* = 86)	Evaluation of the impacts of a team-based learning (TBL) on self-regulated online learning outcomes in a blended under-graduate nursing research and EBP course.	Quasi-experimental, pre- and post-test study design with a control group	**CG: traditional instructor-led (IL)** **EG: Intervention TBL** The same online lessons were used for both the IL control semester and the TBL intervention semester. Students were expected to complete the weekly online content prior to coming to class. TBL classrooms utilize little face-to-face instructor lecture time. Pre-class assignments contain the foundational subject matter that the students are expected to learn prior to coming to class. Student accountability for class preparation is supported by the Readiness Assurance Process^®^ (RAP) which includes a short pre-test, taken by individual students, at the beginning of class. Directly following the individual readiness pre-test, the groups discuss each question on the pre-test and reach agreement on the correct answer. The instructor then discusses pre-test questions with the entire class, providing feedback on the rationale for each correct response and answering any remaining questions students may have.	**Self-regulated learning** was measured by the amount of time that the student spent participating in pre-class online learning activities calculated, by the University Learning Management System, and included multiple viewing times **Cognitive learning outcomes:** Course examination scores. Mean scores from two 50-question multiple choice examinations.	Online viewing time (in seconds) Percent total time Control Group: 42.1% (SD: 32.1%) Experimental Group: 72.0% (SD:34.2%); *p* < 0.001 Examination Scores; range 0–1 (mean; SD) Control Group:0.75; 0.071 Experimental Group: 0.78; 0.071; *p* = 0.003. The mean examination score for the TBL group was 3.32% points higher than the mean examination score for the IL group.	7
Leach et al. (2016) [33]	Australia, 2014 third-year nursing students enrolled in a Bachelor of Nursing program (*n* = 33)	To measure the impact of an undergraduate research education program on undergraduate student nurse attitude, skill and uptake of EBP (the STEP project).	Quasi-experimental, pre- and post-test study design without a control group.	A 16-week research education program, participants: Participation in weekly face-to-face tutorials/online activities is complemented by a series of recommended readings and formative assessments to extend and consolidate student learning. **The first course, ‘Foundations of Research’**. **The second course, ‘Nursing Project’**, is linked to and follows on from the first course. This course builds upon the critical appraisal of the literature in the context of appraising the literature to answer practice-based research questions (as developed in the first course).	**Self-report questionnaire:** **Student nurse Attitude, Skill and Use of EBP, Barriers and facilitators of EBP uptake:** The Evidence-Based Practice Attitude and Utilization Survey (EBASE) This 82-item instrument demonstrates good internal consistency, content validity and acceptable test–retest reliability (Leach & Gillham 2008) [49].	**EBP Attitude** (median): Pre = 33; Post = 33; *p* = 0.238 **EBP-related skills** (median) Pre = 38;Post = 43; *p* < 0.001 **Use of EBP** (median) Pre = 5; post = 7; *p* = 0.002 **Barriers and Facilitators to EBP uptake** Not statistic differences in most of the items. It shows data item by item.	2
Reid et al. (2017) [36]	UK, 2014–2015 Undergraduate nursing and midwifery students Pretest = 124 Posttest = 56	The evidence-based practice was evaluated at the start and the end of the first year of nursing studies within a School of Nursing and Midwifery	Quasi-experimental, pre- and post-test study design without a control group	The module operated during the first year of studies. Different methods such as lectures, small groups, online, etc., were used.	**Self-report questionnaires:** Evidence Based Practice Beliefs Scale (16 items) Evidence Based Practice Implementation Scale. (18 items)	**Results** are showing item by item. Compares pre and post-test groups, not as repeated responses. **Evidence Based Practice Beliefs Scale:** In seven out of sixteen categories were found significant statistical differences. **Evidence Based Practice Implementation Scale:** 13 out of 18 categories.	1
Mena-Tudela et al. (2018) [30]	Spain, 2013–2015 Second-year nursing students (*n* = 83)	The effectiveness of an educational program on the knowledge, skills and attitudes of evidence-based practice was evaluated.	Quasi-experimental, pre- and post-test study design without a control group (pre and 2 post measures)	Intervention: Cross-curricular EBP programme that was consisted of four hours in total (two theory and two computer lab session).	Self-report questionnaire: **Evidence-Based Practice Competence Questionnaire (EBP-COQ)**	**Attitude toward EBP: (score 1–5)** mean; 95% Pre: 3.47; 3.42–3.56 Post1: 3.55; 3.51–3.66 Post2: 3.55; 3.49–3.68 **EBP Knowledge: (score 1–5)** mean; 95% Pre: 2.82; 2.70–2.91 Post1: 3.36; 3.29–3.46 Post2: 3.45; 3.41–3.50 **EBP Skills: (score 1–5)** mean; 95% Pre:2.94; 2.85–3.00 Post1: 2.92; 2.86–3.01 Post2: 3.02; 2.90–3.05 **Global EBP Competence: (score 25–125)** Pre:79.83; 78.63–81.03 Post1: 84.53; 83.23–85.83 Post2: 84.91; 83.26–86.55 *p* < 0.001	4
Oh et al. (2019) [43]	Republic of Korea, Senior nursing students. EG = 21 CG = 24	The effectiveness of an EBP education program was evaluated.	Quasi-experimental, pre- and post-test study design with a control group	**Intervention:** 30 h over five days during the summer vacation period was provided to the intervention group. In the EBP program, six modules were delivered as lectures. A group project with PBL was done with clinical scenarios. CBL was conducted to teach searching strategies of evidence on online databases. TBL was used in teaching “nursing study design.” Two EBP professionals (a school librarian and a clinical expert) were invited as special lecturers on searching databases and the application of EBP in hospitals.	**Self-administered questionnaire** **Level of EBP knowledge (score 0–12):** Utilized the Knowledge of Research Evidence Competencies (K-REC) instrument (Lewis et al., 2011) [50] **EBP self-efficacy (score 26–260)** SE-EBP scale, Korean version (Oh et al., 2016) [51]. **Evidence utilization (score 10–40):** questions from the Knowledge, Attitude, and Behavior (KAB) Questionnaire for EBP (Johnston et al., 2003) [46]	**EBP knowledge** EG: t0 (M = 4.61, SD = 1.19) to t1 (M = 8.44, SD = 1.24) CG: t0 (M = 4.35, SD = 1.76) to t1 (M = 3.95, SD = 1.84); *p* < 0.001 **EBP self-efficacy** EG: t0 (M = 165.05, SD = 45.31) to t1 (M = 221.76, SD = 25.56) CG: t0 (M = 170.96, SD = 35.71) to t1 (M = 165.96, SD = 31.96); *p* < 0.001 **Effects on level of evidence utilization** EG: t0 (resource utilization: M = 15.10, SD = 2.26; to t1 (M = 17.10, SD = 1.76; CG: resource utilization t0 (M = 15.17, SD = 2.22) to t1 (M = 15.38, SD = 2.12). *p* = 0.009 EG: database utilization: t0 (M = 10.90, SD = 1.90) t1 (M = 12.38, SD = 1.47); CG: database utilization t0 (M = 10.71, SD = 1.94) to t0 (M = 10.50, SD = 1.64; *p* = 0.006	5

Acronyms: Evidence based practice (EBP); focused interactive teaching—E-FIT; Knowledge, Attitudes and Behavior—KAB; Team-based learning—TBL; Evidence-based research—EBR.
